# Service Evaluation of Activities Offered in Waterton Ward; Rehabilitation Ward, Medium Secure Forensic Inpatient Unit, Newton Lodge (The Yorkshire Centre for Forensic Psychiatry), Wakefield

**DOI:** 10.1192/bjo.2026.11530

**Published:** 2026-06-30

**Authors:** Julius Akhetuamhen

**Affiliations:** South West Yorkshire Partnership Teaching NHS Foundation Trust, Wakefield, United Kingdom

## Abstract

**Aims:**

- To provide patient with a method to give honest and protected feedback around activities offered on the ward and also to allow the improvement of activities offered on Waterton Ward, Newton Lodge.
- To provide staff with the opportunity to identify gaps in service provision around activities offered on Waterton Ward, Newton Lodge.

**Methods:**

A survey was conducted in person with patients on Waterton ward using a printed survey questionnaire between May and June of 2025. An anonymous staff survey questionnaire was printed and given to members of staff on the ward to complete between May and June 2025

The data were analysed by the project lead. Both quantitative and qualitative data were collected. Data were grouped into themes to aid the analysis and presentation of the results

**Results:**

- The survey highlighted a disparity in awareness of available activities on the ward. While some activities, such as the **Horticulture Garden**, **Community Sessions,** and **Walking Group,** have full awareness among patients, others, such as the **Lego Group** and **Driving Skills Group**, have very low awareness.
- Enjoying the activities, **staff persistence, more awareness, and an additional occupational therapist** are key motivators for engagement.
- Patients expressed interest in additional activities, including **vocational skills like painting and decorating, music groups, more gym time, and more frequent walking groups.**
- The responses emphasize a wide range of life skills patients believe will prepare them for reintegration into the community. Cooking, budgeting, shopping, banking, and IT skills are frequently mentioned, reinforcing the importance of practical education and independence.
- Patient satisfaction with current activities on the ward appears varied–while some patients feel satisfied, a portion remain indifferent or dissatisfied

From the staff survey, **staffing shortages** was consistently mentioned across different staff roles as a barrier to providing rehabilitation services on the ward. Staff satisfaction with the ward activities was mixed with 55% of respondents expressing dissatisfaction.

**Conclusion:**

The findings from the patient survey underscore a need for better communication and promotion of existing activities, improved scheduling, and staff availability. This project highlighted the need for patient and staff participation in improving the interventions and activities offered on the ward.

